# Trocar-assisted, flanged sutureless scleral-fixated intraocular lens implantation combined with silicone oil injection after penetrating keratoplasty surgery

**DOI:** 10.3205/oc000130

**Published:** 2020-02-14

**Authors:** Remzi Karadag, Guler Kilic, Aylin Ardagil, Ahmet Demirok

**Affiliations:** 1 Department of Ophthalmology, Istanbul Medeniyet University School of Medicine, Goztepe, Istanbul, Turkey; 2 Department of Ophthalmology, Istanbul Medeniyet University Goztepe Research and Training Hospital, Goztepe, Istanbul, Turkey

**Keywords:** intraocular lens, silicone oil, sutureless, sutureless intrascleral fixation, trocar, sutureless sclera fixation

## Abstract

We describe a combined technique of trocar-assisted sutureless scleral-fixated intraocular lens implantation and silicone oil injection at the same session. Two 3 mm scleral tunnels were created 2 mm away from and parallel to the limbus with the 23-gauge vitrectomy trocars entering the sclera transconjunctivally at an angle of approximately 10° at the 3 o’clock and 9 o’clock meridians. After the 3-piece foldable IOL was delivered to the anterior chamber through the corneal incision, the tip of one of the IOL haptics was grasped with a 23-gauge serrated retinal forceps entered through the trocar located at the 3 o’clock meridian. Then the haptic was removed from the scleral tunnel together with the trocar out of the globe. The same procedure was applied to the other haptic. A transconjunctival secure 10-0 nylon suture was placed at the scleral tunnel entry site around the haptic. The ends of the haptics were cauterized to make a flange. The resultant flanges of the haptics were pushed back and fixed into the scleral tunnels. Perfluorooctane was taken out of the eye with vitreoretinal surgery and the silicone was injected into the eye to prevent hypotonia. No complications were seen intraoperatively or postoperatively. After 1-month follow-up period, IOL was seen stabilized.

## Introduction

In patients with inadequate capsular support, there are many methods for intraocular lens (IOL) implantation. These include anterior chamber IOL implantation, iris-fixated IOL, and scleral-fixated IOL implantation techniques [1]. Each of these methods has its specific advantages and disadvantages [1]. Sutureless scleral fixation (SSF) IOL implantation methods have recently become popular due to suture-related complications such as inflammation, infection, and suture break or degradation, which results in IOL dislocation [1], [2], [3]. Many different techniques have been described for sutureless scleral IOL fixation [3], [4], [5]. After obtaining successful results with SSF techniques, now these techniques have begun to be applied in a combined surgery [6], [7], [8]. Yet, there are few publications in the literature about this technique combined with other surgeries, these being only case reports [6], [7], [8].

In this article, we aimed to present the operation of SSF IOL implantation and silicone oil injection at the same session due to postoperative hypotonia and aphakia in a patient who had previously undergone keratoplasty because of corneal opacification due to toxic anterior segment syndrome (TASS). 

## Case description

**Figure 1:**
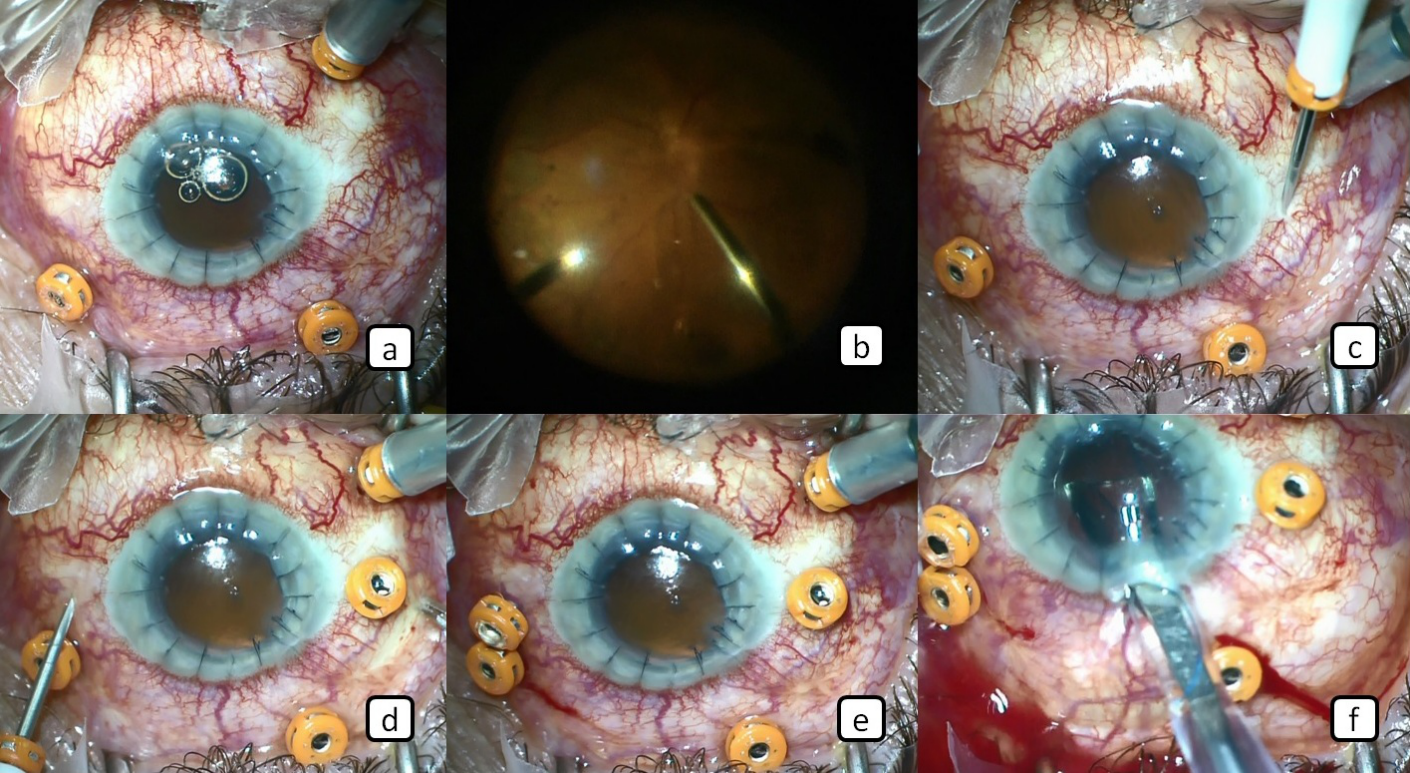
a) Three trocars are applied using a 23-gauge vitreoretinal trocar system for routine vitreoretinal procedure. b) The perfluorooactane is given. c-e) Two 3-mm scleral tunnels are created 2 mm away from and parallel to the limbus with the 23-gauge vitrectomy trocars at the 3 o’clock and 9 o’clock meridians. f) The 3-piece of foldable IOL is delivered to the anterior chamber.

**Figure 2:**
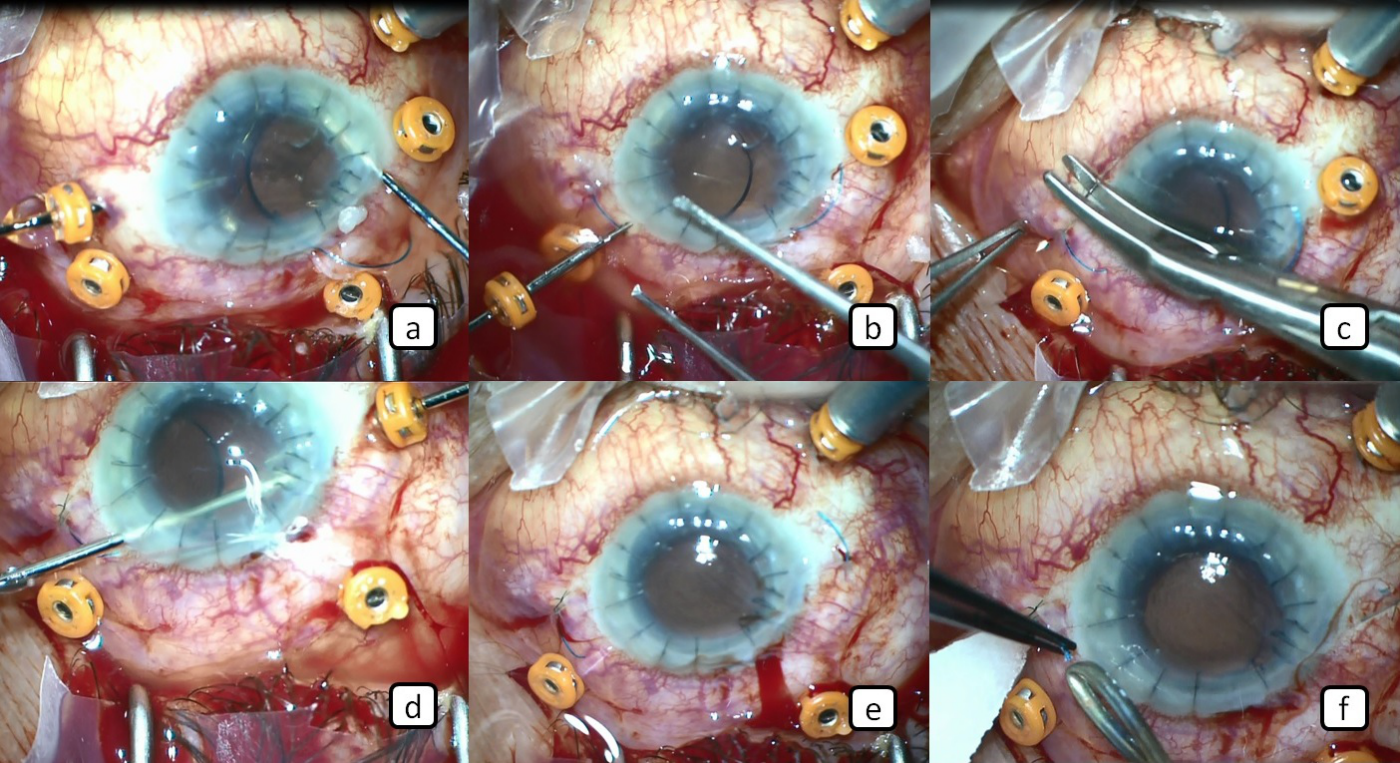
a) The tip of one of the IOL haptics is grasped with a 23-gauge serrated retinal forceps. b) The haptic is removed from the scleral tunnel together with the trocar out of the globe. c) A transconjunctival safety 10-0 nylon suture was placed at the scleral tunnel entry site. d) The tip of the other haptic is grasped a forceps. e) Appearance of the haptics that are removed from the scleral tunnel. f) The end of the haptic is cauterized to make a flange.

**Figure 3:**
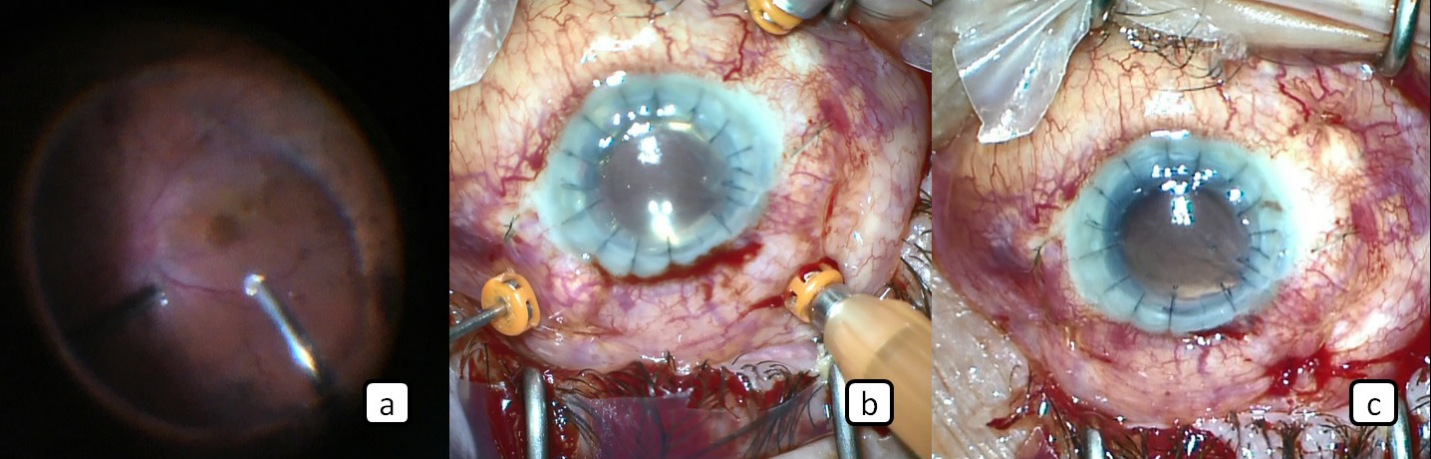
a) The perfluorooactane is removed. b) The silicone oil is given into the vitreous cavity. c) At the end of the surgery.

A 51-year-old male patient who underwent cataract surgery previously in another center developed retinal detachment in the same eye approximately 24 months after the cataract surgery and retinal detachment surgery was performed in our clinic. After this surgery, TASS developed in the patient and corneal opacification and intense membranes occurred around the iris, ciliary body, and intraocular lens despite extensive treatment. This was followed by penetrating keratoplasty. The cyclitic membranes were cleaned and IOL extraction was performed. Postoperatively, the corneal graft was transparent and the visual acuity level of the patient was hand motion. Since our patient had hypotonia for 4 months following surgery as a result of ciliary body shut down, we planned the implantation of trocar-assisted SSF IOL and silicone oil injection at the same session. Surgery was performed as briefly described below: Three 23-gauge vitreoretinal trocars were placed in the pars plana at 2, 8 and 10 o’clock (Figure 1a (Fig. 1)). Then perfluorooactane was given (Figure 1b (Fig. 1)) and appropriate intraocular pressure (IOP) was obtained. Two 3 mm scleral tunnels were created 2 mm away from and parallel to the limbus with the 23-gauge vitrectomy trocars entering the sclera transconjunctivally at an angle of approximately 10° at the 3 o’clock and 9 o’clock meridians and entered into the posterior chamber (Figure 1c–e (Fig. 1)). After the 3-piece foldable IOL was delivered to the anterior chamber through the corneal incision (Figure 1f (Fig. 1)), the tip of one of the IOL haptics was grasped with a 23-gauge serrated retinal forceps entered through the trocar located at the 3 o’clock meridian (Figure 2a (Fig. 2)). And then the haptic was removed from the scleral tunnel together with the trocar out of the globe (Figure 2b (Fig. 2)). After these procedures, a transconjunctival safety 10-0 nylon suture was placed at the scleral tunnel entry site (Figure 2c (Fig. 2)). The same procedure was applied to the other haptic (Figure 2d,e (Fig. 2)). The ends of the haptics were cauterized to make a flange (Figure 2f (Fig. 2)). The resultant flanges of the haptics were pushed back and fixed into the scleral tunnels. After completion of these procedures, perfluorooctane-air, air-silicone exchange was performed (Figure 3a,b (Fig. 3)) to prevent hypotonia. The trocars placed were removed and 3 trocar entries were sutured with 7/0 vycril suture and the operation was terminated (Figure 3c (Fig. 3)). The safety 10-0 nylon sutures were removed 1 week later. It was observed that intraocular pressure was elevated and the visual acuity level of the patient was counting fingers at 1 meter at the first postoperative month.

## Discussion

In case of insufficient capsular support, scleral-fixated intraocular lenses are the method frequently used in patients [1]. One of the most serious sight-threatening complications of IOL implantation with scleral fixation is endophthalmitis and this complication is associated with suture [2]. In the literature, as suture-related complications increase, SSF methods became common [3], [4], [5]. As experience was gained with SSF IOL implantation, the cases in which this method was combined with other surgeries have begun to be published. As the first combined surgery, Prakash and colleagues reported an SSF method using fibrin glue with a femtosecond laser-assisted DSEAK (Descemet’s stripping automated endothelial keratoplasty) surgery in 3 cases [6]. In our previous study, we described the intraocular lens implantation with trocar-assisted SSF combined with penetrating keratoplasty in 4 patients [7]. Afterwards, Sethi and colleagues published a combined surgery of implantation of SSF IOL using a 26-gauge needle in combination with penetrating keratoplasty in 10 cases [8]. In addition to these combined surgeries, we successfully performed SSF surgery with removal of silicone oil from two eyes who underwent vitreoretinal surgery and we observed no complication [9]. In the case presented, we performed surgery of silicone oil injection and trocar-assisted SSF IOL implantation in a case developing hypotonia following surgery of penetrating keratoplasty and cyclitic membrane cleaning performed in the patient with corneal opacification due to TASS occurring after vitreoretinal surgery. To the best of our knowledge, this is the first case in the literature who has undergone surgery in this way. Our patient had undergone penetrating keratoplasty and IOL extraction surgery 2 months ago and he had inadequate capsular support. In patients with insufficient capsule support and who underwent penetrating keratoplasty, the most appropriate method of secondary IOL implantation is still controversial [1], [2], [10]. Scleral fixated IOL lens implantation is more frequently preferred to iris-fixated implantation because of complications such as long learning process, pigment dispersion syndrome, and IOL dislocation [10]. Recently, SSF IOL implantation has begun to be used more often because it does not cause suture-related complications. Since a scleral flap is not created, also the duration of surgery is getting shorter. However, in addition to other complications, this technique has unique complications such as early spontaneous IOL dislocation, persistent hypotony, subconjunctival haptic, and conjunctival erosion due to haptic exposure. Moreover, it needs a learning curve and special tools [3]. 

In conclusion, trocar-assisted SSF method is a suitable surgical method for combination with posterior segment approaches since it is distant from graft endothelium in cases previously having undergone penetrating keratoplasty and because it has few intraocular manipulations and prevents suture-related complications.

## Notes

### Competing interests

The authors declare that they have no competing interests.
